# A high-throughput screen for the identification of compounds that inhibit nematode gene expression by targeting spliced leader *trans*-splicing

**DOI:** 10.1016/j.ijpddr.2019.04.001

**Published:** 2019-04-05

**Authors:** George Cherian Pandarakalam, Michael Speake, Stuart McElroy, Ammar Alturkistani, Lucas Philippe, Jonathan Pettitt, Berndt Müller, Bernadette Connolly

**Affiliations:** a School of Medicine, Medical Sciences and Nutrition, University of Aberdeen, Institute of Medical Sciences, Foresterhill, Aberdeen, AB25 2ZD, UK; b European Screening Centre, University of Dundee, Biocity Scotland, Bo'ness Road, Newhouse, ML1 5UH, Scotland, UK

**Keywords:** Anthelminitics, *Caenorhabditis elegans*, Sinefungin, SL1 *trans*-splicing, HTS assay

## Abstract

Infections with parasitic nematodes are among the most significant of the neglected tropical diseases affecting about a billion people living mainly in tropical regions with low economic activity.

The most effective current strategy to control nematode infections involves large scale treatment programs with anthelmintic drugs. This strategy is at risk from the emergence of drug resistant parasites. Parasitic nematodes also affect livestock, which are treated with the same limited group of anthelmintic drugs. Livestock parasites resistant to single drugs, and even multi-drug resistant parasites, are appearing in many areas. There is therefore a pressing need for new anthelmintic drugs.

Here we use the nematode *Caenorhabditis elegans* as a model for parasitic nematodes and demonstrate that sinefungin, a competitive inhibitor of methyltransferases, causes a delay in development and reduced fecundity, and inhibits spliced leader *trans*-splicing. Spliced leader *trans*-splicing is an essential step in gene expression that does not occur in the hosts of parasitic nematodes, and is therefore a potential target for new anthelmintic drugs.

We have exploited the ability of sinefungin to inhibit spliced leader *trans*-splicing to adapt a green fluorescent protein based reporter gene assay that monitors spliced leader *trans*-splicing for high-throughput screening for new anthelmintic compounds. We have established a protocol for robust high-throughput screening, combining mechanical dispensing of living *C. elegans* into 384- or 1536- well plates with addition of compounds using an acoustic liquid dispenser, and the detection of the inhibition of SL *trans*-splicing using a microplate reader. We have tested this protocol in a first pilot screen and envisage that this assay will be a valuable tool in the search for new anthelmintic drugs.

## Highlights

• GFP-based assay to monitor spliced leader *trans*-splicing in living nematodes.
• Suitable for high throughput screening.
• Nematode spliced leader *trans*-splicing inhibited by sinefungin.

## Graphical abstract

**Figure:**
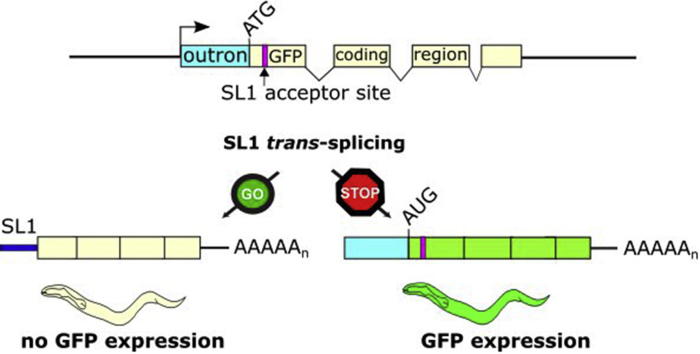

## 1 Introduction

Parasitic nematode infections are the cause of several of the major neglected tropical diseases identified by the World Health Organization that cause long-term disability and can lead to disfigurement (Hotez, 2013). Hookworm infections, ascariasis and trichuriasis are the most prevalent neglected tropical diseases, affecting predominantly “the bottom billion” of the world population living in extreme poverty in regions where these parasites are endemic (Collier, 2008; Hotez, 2013). Frequently, individuals suffer from two or all three of these conditions. There is good evidence that they affect physical and mental development, causing stunted growth and deficits in memory, intelligence and cognition (Crompton, 2000; Bethony et al., 2006). Parasitic nematodes also impact on livestock production, and infect equids and companion animals, with annual treatment costs (not including the loss of production) in the tens of billion dollars worldwide (Roeber et al., 2013).

Currently, the most effective strategy to control nematode infections is the use of anthelmintic drugs. The World Health Organization advocates periodic, community-wide treatment with anthelmintic drugs focusing predominantly on school-age children, which are at particular risk of infection (WHO Resolution 54.19, 2001). This strategy, with treatments using compounds belonging to the benzimidazole class of anthelmintics, has led to clear health and fitness benefits with improvements in physical development and mental abilities (Hotez, 2013).

However, this approach is jeopardised by emerging resistance against anthelmintic drugs. In livestock parasites, resistance against the three long-used major classes of anthelmintic drugs, benzimidazoles, imidothiazoles/tetrahydropyrimidines and macrocyclic lactones, is widespread, and there are even recent reports of resistance against the newest drug, the amino-acetonitrile derivative monepantel (Kaplan and Vidyashankar, 2012; Mederos et al., 2014). Treatment regimes of human populations need to be carefully monitored and managed to minimise the risk of the emergence of drug resistant human parasites (Vercruysse et al., 2011). A recent report of a reduced efficacy of treatment with albendazole raises the suspicion of the emergence of drug resistant *Ascaris lumbricoides* (Krücken et al., 2017). This highlights that there is an urgent need for the identification of anthelmintics with novel mechanisms of action.

**Fig. 1:**
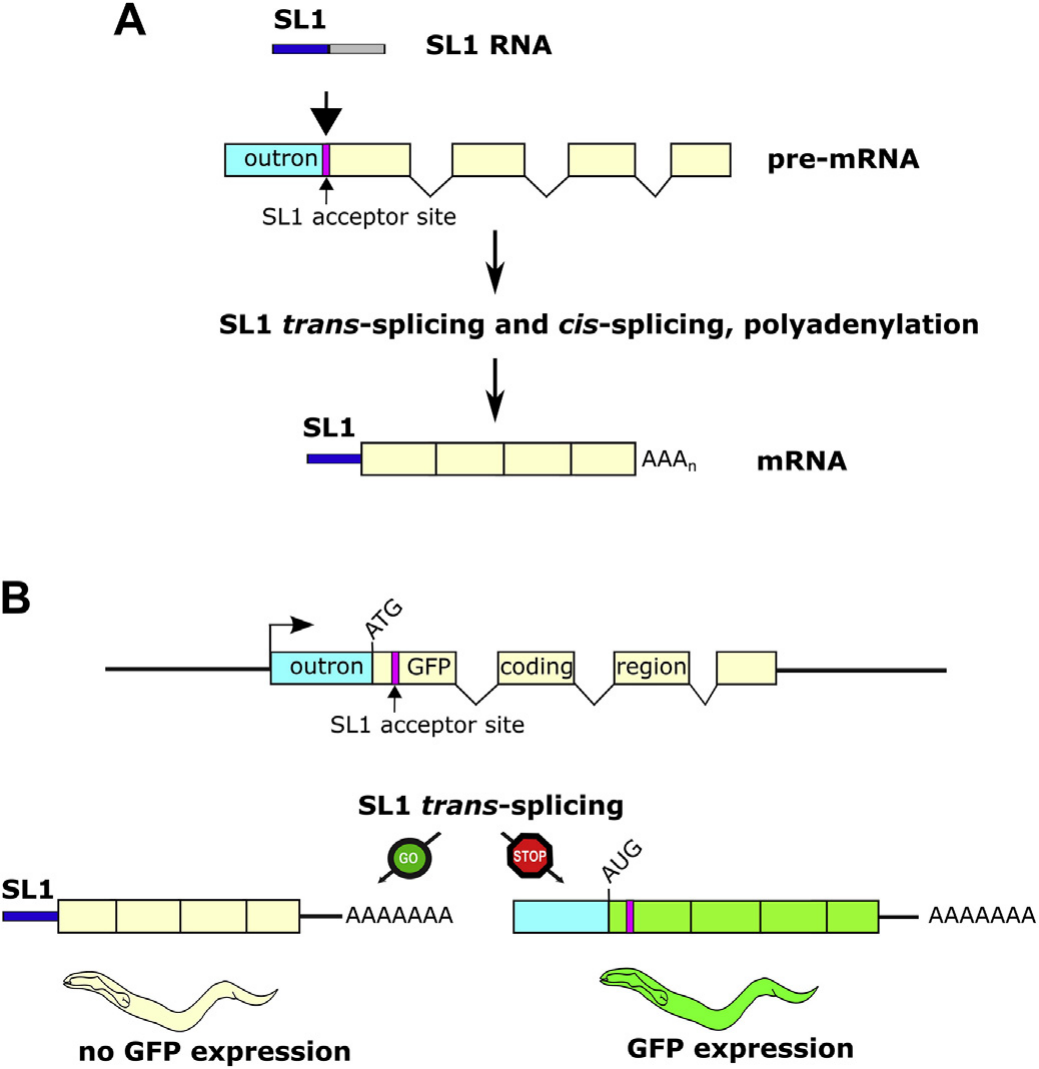
**A. SL1 *trans*-splicing mechanism.** SL1 spliced leader *trans*-splicing is the addition of a specialised exon known as the spliced leader 1 (SL1) to the 5′ ends of pre-mRNA transcripts at the SL1 splice acceptor site, resulting in the removal of the 5′ untranslated region referred to as the “outron”. The spliced leader SL1 is donated by a separate RNA, the SL1 RNA. **B. A GFP based *in vivo* assay that monitors SL1 *trans*-splicing.** Schematic representation of the *gfp* reporter gene with the outron sequence and the SL1 splice acceptor site 3′ of the ATG initiation codon (schematic adapted from (Philippe et al., 2017)). During SL1 *trans*-splicing, the outron sequence is removed from *gfp* mRNA and replaced by SL1, resulting in the removal of the initiation codon and thus preventing synthesis of functional GFP. Inhibition of SL1 *trans*-splicing results in GFP expression, because the start codon is retained in the *gfp* reporter mRNA. Presence and absence of SL1 *trans*-splicing are indicated with GO and STOP signs, respectively.

Here we describe the adaptation of a GFP-based assay that detects the inhibition of spliced leader 1 *trans*-splicing (SL1 *trans*-splicing) in living *C. elegans* for high-throughput screening (Philippe et al., 2017). Spliced leader *trans*-splicing is conserved across the nematode phylum and occurs in cestodes and trematodes, but does not occur in vertebrates or plants (Rajkovic et al., 1990; Brehm et al. 2000, 2002; Lasda and Blumenthal, 2011; Pettitt et al., 2014). SL1 *trans*-splicing involves the transfer of the short SL1 RNA fragment from a non-polyadenylated RNA, called the spliced leader RNA (SL1 RNA), onto the 5’ end of a pre-mRNA molecule (Krause and Hirsh, 1987) (Fig. 1A). Spliced leader *trans*-splicing also requires the U2 snRNP and the U4/U6.U5 tri-snRNP involved in intron removal by *cis-*splicing (Hannon et al., 1991; Maroney et al., 1996).

Importantly, spliced leader *trans*-splicing is an essential function that requires the SL RNA and associated proteins. Animals are most sensitive to the inhibition of SL1 *trans*-splicing during early development when extensive gene expression is required for cell division and differentiation: both loss of SL1 RNA genes and RNAi knockdown of the essential spliced leader *trans*-splicing factor SNA-2 lead to embryonic lethality (Ferguson et al., 1996; MacMorris et al., 2007).

Our GFP-based assay produces functional GFP protein in the intestine of adult animals when SL1 *trans*-splicing is inhibited (Philippe et al., 2017) (Fig. 1B). This allows the detection of inhibition of SL1 *trans*-splicing independent of effects on early development. Based on our investigation of factors involved in SL1 *trans*-splicing, reporter gene activation occurs in response to the depletion of the function of non-essential factors such as SNA-1, whose loss causes cold-sensitive defects in viability, as well as clearly essential factors such as SNA-2, indicating that the assay can be used for the detection of compounds that inhibit SL1 *trans*-splicing to such a degree that they cause lethality (Philippe et al., 2017).

The reporter system was designed to detect factors whose inhibition preferentially interferes with SL1 *trans*-splicing. The GFP reporter gene contains three introns; any compound with a severe effect on *cis*-splicing should not lead to reporter gene activation as intron inclusion generates non-functional transcripts containing premature termination codons. However, we envisage that as part of the characterisation of any inhibitors of SL1 *trans*-splicing additional tests will need to be done to specifically rule out any effects on *cis*-splicing of host transcripts.

Here, we use this assay to demonstrate that sinefungin inhibits SL1 *trans*-splicing. Sinefungin is a naturally occurring nucleoside with similarity to S-adenosyl-1-methionine (Barbés et al., 1990), and acts as an inhibitor of methyltransferases involved in the methylation of nucleic acids and proteins (Vedel et al., 1978; Borchardt et al., 1979).

We have exploited the ability of sinefungin to inhibit SL1 *trans*-splicing to adapt our GFP-based assay for liquid-based, semi-automated identification of novel anthelmintic compounds *via* high-throughput screening (HTS). The HTS assay combines automated liquid dispensers for the addition of nematodes and compounds into 384- or 1536-well plates, with the subsequent detection of GFP production following SL *trans*-splicing inhibition using a microplate reader. The assay was developed to provide suitable robustness and reproducibility for HTS, as defined by Z′-factor values of ≥0.5 (Zhang et al., 1999). We have executed a pilot screen, demonstrating the feasibility of HTS, and test the effects of some key modification on the robustness of the assay.

## 2 Materials and methods

### 2.1 Sinefungin and isoginkgetin

Sinefungin was purchased from Enzo Life Sciences (ALX-380-070-M005). A 40 mM sinefungin stock solution was prepared in DMSO (Sigma-Aldrich, 276855). Lower concentrations were prepared by serial dilution in DMSO and stored at −20 °C until required. Isoginkgetin was purchased from Merck (416154-10) and dissolved at 40 mM in DMSO. Working stocks of 1 mM, 5 mM and 20 mM were prepared by dilution in DMSO.

### *2.2* *C. elegans* strains

Strains used were PE796 [*fem-1(hc17) IV; feIs14 [P_vit-2_*::*outron*::*gfp*^*M1A*^ *P_myo-3_*::*mCherry] V*] and PE797 [*fem-1(hc17) IV; feIs14 [P_vit-2_*::*outron*::*gfp*^*M1A*^ *P_myo-3_*::*mCherry] V; bus-8(e2698) X*]. The *C. elegans* strains were maintained on NGM agar plates with a lawn of *Escherichia coli* strain OP50 (Brenner, 1974; Stiernagle, 2006) in a temperature-controlled incubator at 20°C.

### 2.3 Development and egg viability assays

Approximately 50 eggs or 20 synchronous L1 larvae were applied onto 3 cm NGM agar plates with or without 1% DMSO, or with 1% DMSO and 400 μM sinefungin, and seeded with an OP50 lawn (Stiernagle, 2006). Development was monitored at 12–15 h intervals for up to 80 h  at 25°C. To test egg viability, single worms were added to ten 3 cm NGM agar plates with or without 1% DMSO, or with 1% DMSO and 400 μM sinefungin, and incubated at 25°C. Adults were moved to fresh plates every 12 h (plates with/without DMSO) or 16 h (plates with sinefungin/DMSO), and eggs were left to hatch at 25°C. The numbers of eggs and hatched eggs were recorded.

### 2.4 Preparation of bacteria for feeding

Concentrated pellets of OP50 bacteria were prepared as described and stored at −20°C (Stiernagle, 2006).

### 2.5 Liquid culture of *C. elegans*

Age-synchronous *C. elegans* cultures were produced by treatment of gravid animals from 4 to 5 10 cm petri plates with hypochlorite solution (Stiernagle, 2006). Eggs were resuspended in 120 ml M9 buffer supplemented with 1% LB broth (Leung et al., 2011), transferred into a 1 L spinner flask (Corning 4500-1L) and grown under constant stirring at 200 rpm at 25°C. Alternatively, the eggs were resuspended in 25 or 30 ml M9 buffer supplemented with 1% LB broth and transferred into 250 ml or 500 ml sterile flask, and grown at 25°C in a temperature-controlled orbital shaking incubator at 160 rpm.

After overnight incubation, L1 larvae were collected by centrifugation and resuspended at a density of 2000–2500 animals/ml in 120 ml S-complete medium supplemented with 16–18 mg/ml OP50 *E. coli* (Stiernagle, 2006). Animals were grown to adults in a 1 L spinner flask stirred at 200 rpm at 25°C. Alternatively, 25 ml or 30 ml cultures were grown in 250 ml or 500 ml glass flasks at 25°C in a shaking incubator. Animals were allowed to settle under gravity at room temperature for 10 min (Leung et al., 2011). Then the animals were washed once in S-complete medium and then resuspended in S-complete medium supplemented with 4–6 mg/ml OP50 *E. coli* at a density of 1000–1250 animals/ml. Alternatively, animals were washed twice in S-complete medium and then resuspended at a density of 1000–1250 animals/ml.

### 2.6 High-throughput screening

For dispensing the animals into 384- or 1536-well plates, 40 ml C*. elegans* suspension at a density of 1000–1250 animals/ml prepared as described under 2.5 above was transferred into a sterile 50 ml glass bottle with a magnetic stirrer bar and stirred at 80 rpm at room temperature. Animals were dispensed into 384- or 1536-well plates using a Multidrop™ Combi Reagent Dispenser (Thermo Scientific 84030) with a standard dispensing cassette (Thermo Scientific 24072670) using default settings. Where indicated, a Matrix WellMate™ (Thermo Scientific 201-10001) with a 8-channel standard bore tubing cartridge (Thermo Scientific 201-30001) was used instead. Equipment was sterilised and rinsed with 70% ethanol and then sterile water before use. The number of animals dispensed per well was tested using clear bottom 384-well plates (Greiner Bio-One 781097) and if necessary the density of animals was adjusted to dispense 20-25 animals in 20 μl into 384-well plates or 5–10 animals in 10 μl into 1536-well plates. For screening, animals were dispensed into black opaque 384-well plates (Greiner Bio-One, 784076) or 1536-well plates (Greiner Bio-one, 783076).

Sinefungin dissolved in DMSO was added to final concentrations of 400 μM sinefungin/2% DMSO, and 800 μM sinefungin/2% DMSO using an acoustic liquid dispenser (ECHO 550 from Labcyte, Inc). Treatment with 2% DMSO only was done as a control. The plates were kept in closed plastic containers humidified by including tissue soaked with distilled water and incubated at 25°C. GFP fluorescence was measured using a PHERAstar FS microplate reader (BMG Labtech, Inc) with excitation/emission wavelengths of 485/520 nm in top reading mode. Samples were normally measured using the orbital averaging mode with 25 flashes per read.

Assay robustness and reproducibility were tested by calculating the Z′-factor (Zhang et al., 1999) and the signal to background (S/B) ratio using equations (1), (2) shown below, using the mean (μ) and standard deviation (σ) of the GFP signal from sinefungin-treated (P) and DMSO only treated animals (N). The standard deviation of S/B was approximated using formula (3).

(1) $$Z^{'}=1-\left(\frac{3\times \sigma P+3\times \sigma N}{\mu P-\mu N}\right)$$

(2) $$\frac{S}{B}=\frac{\mu P}{\mu N}$$

(3) $$\sigma \frac{S}{B}≅\sqrt{{{\left(\frac{\sigma P}{\mu P}\right)}^{2}+\left(\frac{\sigma N}{\mu N}\right)}^{2}}$$

### 2.7 Drug library screening

A library of 5195 structurally diverse drug-like compounds was obtained from the BioAscent Compound Cloud (www.bioascent.com/compoundcloud/, BioAscent, Biocity Scotland, Newhouse, Scotland). In addition, 727 compounds from the NIH Clinical Collection and 853 compounds custom selected from the SelleckChem screening libraries collection were tested. Screening was done in 384-well plates. On each plate, PE796 animals were also treated with 800 μM sinefungin/2% DMSO or with 2% DMSO. Animals were exposed to compounds at a concentration of 50 μM in the presence or absence of OP50 bacteria, and fluorescence was captured after 4 h using a PHERAstar FS microplate reader as described above. Z′-factor and signal to background ratios were determined using ActivityBase software (IDBS) and data normalisation was done using Vortex software (dotmatics). Compounds reaching at least 20% effect relative to control treated animals (sinefungin/DMSO control treatment is 100% effect, DMSO only control treatment is 0% effect) were selected for further analysis. Dose-response assays were performed using concentrations between 0.08 μM and 80 μM sinefungin. GFP fluorescence was measured using a PHERAstar FS microplate reader after 4 h and analysed using IDBS ActivityBase software.

### 2.8 Fluorescence microscopy

GFP intensity was measured as described (Philippe et al., 2017). Briefly, 10 animals were immobilized using 0.5% l-phenoxy-2-propanol on slides with 1% agarose cushions and covered with a cover slip. GFP and mCherry fluorescence were captured using a Zeiss Axioplan 2 fluorescence microscope and analysed using ImageJ software (Schneider et al., 2012). The GFP fluorescence intensity of each animal was normalised with respect to its mCherry fluorescence intensity. Statistical analysis was done using Microsoft Excel 2013 or GraphPad Prism version 5.00 for Windows, GraphPad Software, San Diego, CA, USA.

### 2.9 Reverse transcription-quantitative PCR (RT-qPCR)

Total RNA was prepared as described (Philippe et al., 2017). Briefly, 1 ml of liquid culture containing 750 - 1000 adults were collected and washed once in 1 ml M9 buffer. Then total RNA was prepared using the PureLink RNA Mini kit (Life Technologies) with modifications for TRIzol treated samples and DNase treatment as described by the manufacturer. For cDNA synthesis, total RNA was reverse transcribed using oligo(dT) primers and M-MLV Reverse Transcriptase, according to the manufacturer's instructions (Promega). qPCR assays are described in detail elsewhere (Philippe et al., 2017). Briefly, qPCR assays were designed using the Universal Probe Library Assay Design Centre (Roche) and were done in three technical replicates on a Roche Lightcycler 480 using standard settings.

Primers TAAAATTATCATGTTTTCAGGAC and GTTGCATCACCTTCACCCTC were used to detect the outron of *gfp* transcripts, and primers CCACATGGTCCTTCTTGAGTTT and ATAGTTCATCCATGCCATGTGTA were used to detect the last *gfp* exon (internal *gfp* amplicon). Primers TATATTTCTTGTGTTTTGTTCGGATT and ACGGCCTTCTTCTTCTTGGT were used to detect the outron of *rps-3* transcripts, and primers TCCTTCCAAAGGAACCACAC and TGCTGGGACTTGAACATCCT were used to detect the last exon of *rps-3* transcripts (internal *rps-3* amplicon).

Data was analysed using the comparative C_T_ method (Schmittgen and Livak, 2008). Outron levels were standardised with respect to the levels of the internal RNA amplicon. Outron levels detected in untreated animals were defined as 1.

## 3 Results and discussion

### 3.1 Sinefungin inhibits spliced leader trans-splicing in nematodes

We have generated a *gfp* reporter gene assay that detects the inhibition of SL1 spliced leader *trans*-splicing in living *C. elegans* (Fig. 1). The reporter gene is expressed under the control of the *vit-2* promoter, limiting its expression to the intestine of L4 and adult hermaphrodite animals (Kimble and Sharrock, 1983; MacMorris et al., 1992). *vit-2* promoter controlled expression of the *gfp* reporter gene allows the identification of factors that inhibit SL1 *trans*-splicing in fully developed animals, without complications in assay interpretation caused by effects on early development, which is particularly sensitive to the loss of spliced leader *trans*-splicing (Ferguson et al., 1996). Ultimately, we expect active compounds to interfere with nematode development, but this will need to be tested independently.

Normally, the *gfp* initiation codon located upstream of a splice acceptor site is removed by SL1 *trans*-splicing, resulting in a transcript with a truncated open reading frame unable to produce functional GFP (Philippe et al., 2017) (Fig. 1B). The inhibition of SL1 *trans*-splicing allows the production of functional GFP by translation of non-processed, outron-containing mRNA. The *gfp* reporter gene used minimises effects caused by the inhibition of factors shared between SL1 *trans*-splicing and intron removal by *cis*-splicing (Hannon et al., 1991; Maroney et al., 1996). The *gfp* reporter gene contains three introns; any compound inhibiting *cis*-splicing would interfere with the removal of introns and thus generate transcripts with two premature termination codons. Such transcripts are degraded by nonsense-mediated RNA decay (Hug et al., 2016), and, if translated, would create a truncated protein terminated 50 amino acids after the GFP chromophore. The selectivity of the assay for inhibition of SL1 *trans*-splicing is supported by the observation that treatment of PE796 animals carrying the *gfp* reporter gene with 20 μM, 100 μM and 400 μM isoginkgetin, a known inhibitor of *cis*-splicing (O'Brien et al., 2008), did not lead to reporter gene activation (data not shown). However, we cannot rule out that our assay would identify compounds inhibiting both SL1 *trans*-splicing and *cis*-splicing. It will be necessary to confirm specificity for SL1 *trans*-splicing by ruling out inhibition of *cis*-splicing, preferably using a cell-based assay derived from parasite hosts.

Sinefungin, a naturally occurring nucleoside isolated from *Streptomyces griseolus* and *S. incarnates* with structural similarities with S-adenosyl-1-methionine, has antitrypanosomal activity and inhibits SL *trans*-splicing in these organisms (Bachrach et al., 1980; Dube et al., 1983; McNally and Agabian, 1992). The precise mechanism of this inhibition is not known. Sinefungin acts as a competitive inhibitor of methyltransferases involved in the methylation of nucleic acids and its inhibition of spliced leader *trans*-splicing has been linked to an inhibition of the formation of the hypermethylated cap 4 structure of SL RNA (Pugh et al., 1978; McNally and Agabian, 1992). However, sinefungin also inhibits methyltransferases involved in the methylation of proteins, a process required for the assembly of snRNPs (Meister et al., 2001; Zheng et al., 2012). It is therefore possible that sinefungin acts at several levels to inhibit SL *trans*-splicing in trypanosomes.

**Fig. 2:**
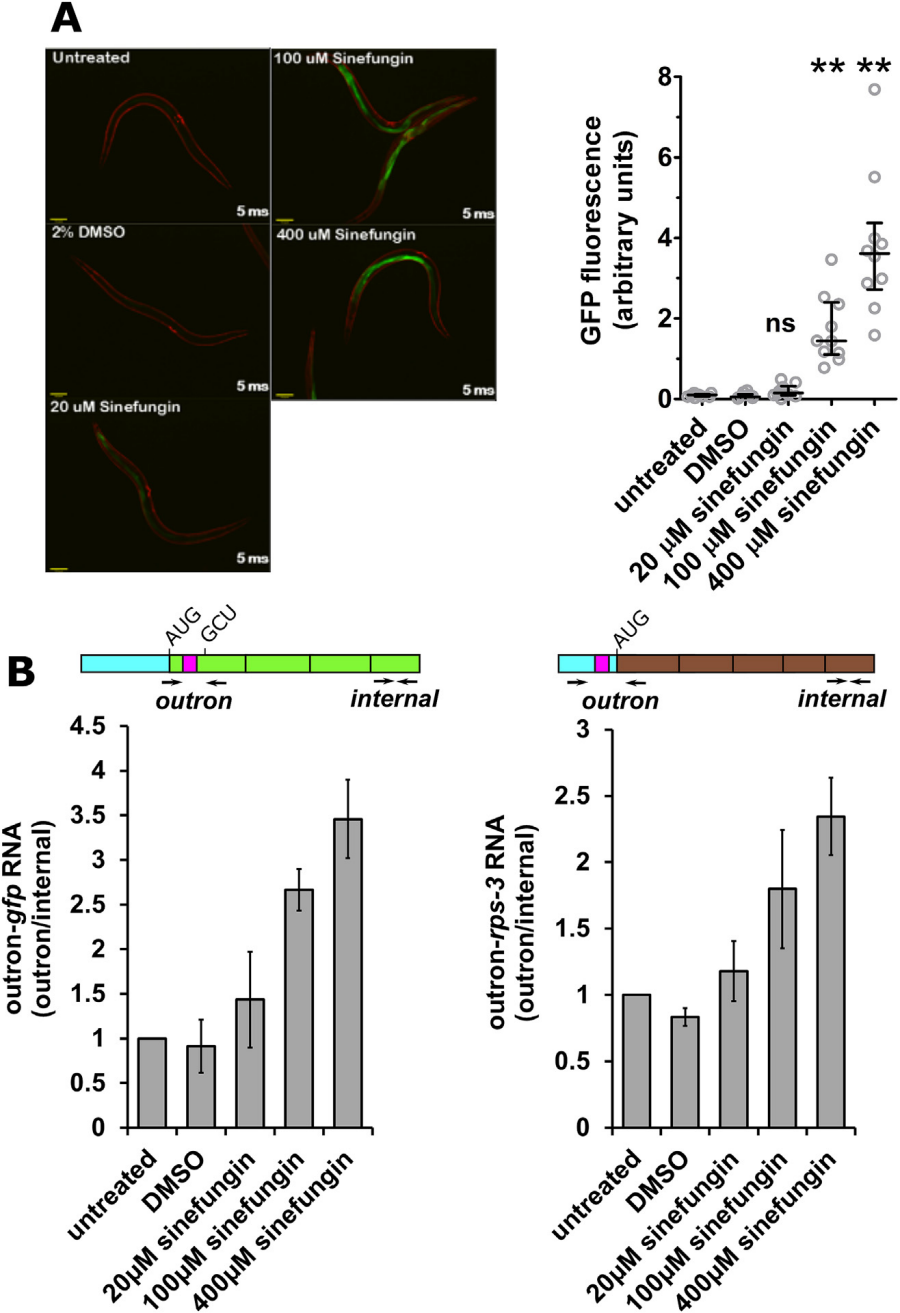
**Sinefungin inhibits SL *trans*-splicing.** A. Exposure to sinefungin activates *gfp* reporter gene expression. PE796 animals in S complete medium supplemented with OP50 bacteria were either left untreated, treated with DMSO (2%) or with 20 μM, 100 μM or 400 μM sinefungin in 2% DMSO for 21 h. *gfp* expression was then analysed by fluorescence microscopy. In the micrographs shown the exposure time was 5 ms and the scale bar represents 100 μm. The graph plots *gfp* expression standardised with respect to mCherry in arbitrary units. 10 animals were analysed for each treatment, shown are the median and first and third quartile. (“ns” indicates values not significantly higher than in untreated and DMSO treated animals (P > 0.05; ANOVA), ** indicates values significantly higher than in untreated and DMSO treated animals (P ≤ 0.01; ANOVA)). B. Treatment with sinefungin inhibits SL *trans*-splicing. PE796 animals were treated with sinefungin for 21 h as described under (A) and then harvested for cDNA synthesis as described in Materials and Methods. SL1 *trans*-splicing of *gfp* reporter transcripts and of endogenous *rps-3* transcripts was analysed by reverse transcription followed by qPCR. The schematic diagrams indicate the location of primers used for PCR on the *gfp* and *rps-*3 RNAs. “outron” indicates the primer pair used to measure the efficiency of SL1 spliced leader *trans*-splicing, and “internal” indicates the primer pair used to measure RNA levels. Outron sequences are shown in blue and the SL1 acceptor sites in pink. *gfp* and *rps-3* open reading frames are shown in green and brown, respectively. The levels of non-*trans*-spliced outron-*gfp* reporter transcripts and of outron-*rps-3* transcripts were standardised with respect to RNA levels measured using the internal primer pairs, and the levels in untreated animals were defined as 1. Note that an increase in outron-*gfp* RNA levels reflects the inhibition of *trans*-splicing. The graphs show the average of three biological replicates, error bars indicate the standard deviation. Inhibition of SL1 *trans*-splicing, as measured by outron retention, increases significantly between treatments with 20 μM, 100 μM and 400 μM sinefungin (P ≤ 0.05; *t*-test). (For interpretation of the references to colour in this figure legend, the reader is referred to the Web version of this article.)

As *C. elegans* SL RNAs are also subject to hypermethylation of the cap structure (Thomas et al., 1988) we examined whether sinefungin could also inhibit SL *trans*-splicing in *C. elegans*, using the PE796 strain carrying a *gfp* reporter gene engineered to produce GFP when SL *trans*-splicing is inhibited (Fig. 2). We observed that exposure to sinefungin caused increased expression of this *gfp* reporter gene. GFP expression was weak but visible in PE796 animals treated with 20 μM sinefungin, and was very clear in treatments with 100 μM and 400 μM sinefungin, compatible with a dose-dependent inhibition of SL1 *trans*-splicing (Fig. 2A).

Since sinefungin was dissolved in DMSO, to rule out that DMSO treatment alone led to reporter gene activation, PE796 animals were treated with 2% DMSO, the maximum DMSO concentration used throughout this study. This did not result in reporter gene activation (Fig. 2A) and, in agreement with observations by others (Katiki et al., 2011), 2% DMSO did not have an obvious detrimental effect on the animals.

RT-qPCR was used to confirm that *gfp* activation was indeed due to a defect in SL *trans*-splicing. To detect the inhibition of *trans*-splicing we measured the levels of outron-containing transcripts using primers targeting the outron regions of *gfp* reporter gene transcripts and of *rps*-3 transcripts (Fig. 2B). *rps-3* is an endogenous gene known to be subject to SL1 *trans*-splicing (Morton and Blumenthal, 2011). The levels of outron-containing transcripts were normalised to amplicons derived from the coding regions of the *gfp* and *rps-3* transcripts, respectively. The proportion of non-*trans*-spliced, outron-containing *gfp* and *rps-3* transcripts increased upon exposure to sinefungin in a dose-dependent manner, confirming that sinefungin inhibits SL *trans*-splicing.

Exposure to sinefungin causes a delay of *C. elegans* development and reduces fecundity. When growing *C. elegans* eggs or synchronised L1 larvae to adults on plates in the absence or presence of 1% DMSO, or of 400 μM sinefungin and 1% DMSO, we observed that exposure to sinefungin led to a 12 h −15 h delay in the appearance of adult animals. Egg laying of animals exposed to sinefungin (400 μM sinefungin, 1% DMSO) was significantly reduced compared to control animals exposed to 1% DMSO only; from an average of 64 eggs laid per animal (n = 10) during a 36 h period to 48 eggs laid per animal (n = 10) during a 48 h period in the presence of sinefungin (*t*-test, p ≤ 0.001). The proportion of eggs hatched was similar for animals treated with sinefungin/DMSO compared to DMSO only treated animals (89% and 87.5%, respectively). Given that drug uptake of *C. elegans* grown on plates is highest in the first 24 h, and that the presence of live bacteria over time significantly reduces the drug concentration in the medium (Zheng et al., 2013), the developmental delay and reduced fecundity observed are most likely a reflection of a mild treatment with sinefungin.

To our knowledge this is the first demonstration of a compound inhibiting SL1 *trans*-splicing in nematodes. The inhibition of SL RNA cap hypermethylation by sinefungin may be responsible for the inhibition of SL *trans*-splicing (McNally and Agabian, 1992). However, as other key steps such as Sm protein assembly also require protein methylation, sinefungin may have a broader effect on SL *trans*-splicing (Meister et al., 2001; Gonsalvez et al., 2007). Furthermore, sinefungin is an inhibitor of a large number of S-adenosyl-1-methionine dependent protein, RNA and DNA methyltransferases (Pugh et al., 1978; Barbes et al., 1990; Zheng et al., 2012). Therefore we cannot exclude the possibility that the inhibition of spliced leader *trans-*splicing, and the detrimental effects on development and egg laying caused by sinefungin, are due to an inhibition of methyltransferases acting in other pathways. We can also not be sure that the inhibition of SL1 *trans*-splicing is the direct cause of the developmental delay and reduced fecundity, because of the effects of sinefungin on a wide range of methyltransferases.

Sinefungin is not a suitable anthelmintic agent because of its toxicity to hosts (Zweygarth et al., 1986). However, one possible route to a new anthelmintic drug would be the identification of sinefungin's key target(s) in spliced leader *trans*-splicing, which in turn would allow the development of less toxic, target-selective derivatives (Zweygarth et al., 1986; Devkota et al., 2014). In the work described here we have used sinefungin as an active compound for high-throughput screening assay development.

### 3.2 Development of a high-throughput screening assay protocol to identify inhibitors of SL1 trans-splicing

The HTS-ready assay was established at the facilities of the European Screening Centre, Newhouse, Scotland. Eggs isolated from gravid PE796 adults were synchronised at L1 stage in liquid culture, and then grown to adult stage and used for assay development. Adult PE796 animals were used because the *vit-2* promoter in the reporter construct is active in late L4/adult stage of transgenic animals (Kimble and Sharrock, 1983; MacMorris et al., 1992; Philippe et al., 2017). Animals were dispensed into black opaque 384-well plates with round wells with a high base (Greiner Bio-One, 784076). These plates were used to minimise interference from background fluorescence or from fluorescence from other wells (Jones et al., 2012) and the conical geometry facilitates reading of the wells from the top of the plate.

**Fig. 3:**
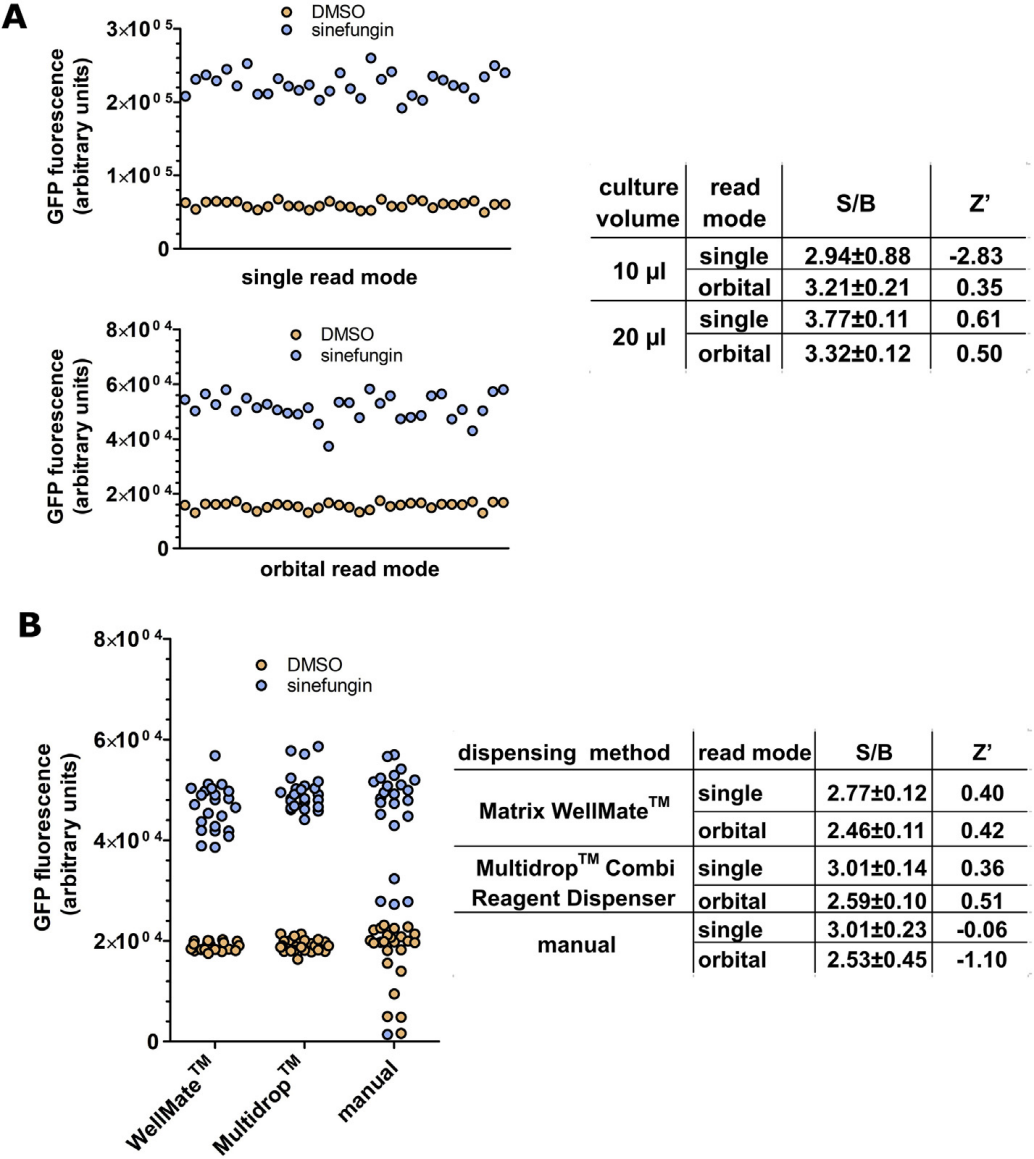
**Culture volume, read mode, and dispensing affect high-throughput screen robustness**. A. Culture volume and read mode. 20–25 animals were manually transferred into wells of a 384-well plate in either 10 μl or 20 μl of S-complete medium containing OP50 bacteria. Animals were then exposed to 400 μM sinefungin/2% DMSO (32 wells) or with 2% DMSO only (32 wells), using an ECHO 550 acoustic liquid handler to dispense the compounds. Fluorescence was measured after 21 h using either single read or orbital averaging mode of a PHERAstar FS microplate reader. The graphs show fluorescence measurements taken in either single read or orbital averaging mode for 20 μl cultures for the treatments with sinefungin/DMSO or with DMSO only. The table shows signal/background ratios (S/B) with standard deviations and Z′-factors from the analysis using orbital averaging mode and single read mode for both culture volumes, calculated as described in Materials and Methods. B. Dispensing method and read mode. 20–25 animals were transferred into wells of a 384-well plate in 20 μl of S-complete medium containing OP50 bacteria using the methods indicated. Animals were then treated with 400 μM sinefungin/2% DMSO (32 wells) or with 2% DMSO only (32 wells). Fluorescence was measured after 21 h using a PHERAstar FS microplate reader using either single read or orbital averaging mode. The graph shows fluorescence measurements of the treatments with sinefungin/DMSO or with DMSO only taken in the orbital averaging mode. The table shows signal/background ratios (S/B) with standard deviations and Z′-factors for measurements taken in single read and orbital averaging mode.

GFP fluorescence resulting from the activation of the reporter gene by inhibition of SL *trans*-splicing was measured using a PHERAstar FS microplate reader. We first compared the effect of the culture volume by manually dispensing animals in either 10 μl or 20 μl of medium (Fig. 3A) followed by addition of sinefungin in DMSO or DMSO only using an acoustic liquid dispenser (Echo 550, Labcyte, Inc.). As the live animals are in constant motion in liquid media, we decided to compare two signal reading modes of the PHERAstar FS microplate reader: repeat measurements at a single point at the centre of each well; or orbital averaging where measurements are taken along a defined orbit. The signal-to-background (S/B) ratios (equations (2), (3)) comparing *gfp* fluorescence in sinefungin treated animals with control DMSO- treated animals were between 2.94 and 3.77 (Fig. 3A), indicating largely similar *gfp* reporter gene activation measured independent of read mode and culture volume. Importantly, the S/B ratios also reflect the activation of *gfp* reporter gene expression observed by microscopy and RT-qPCR in Fig. 2.

To assess the suitability of the assay for HTS, the Z′-factor was calculated for each condition (Zhang et al., 1999). A Z′-factor value of 0.5 or above indicates clear distinction between positive signals and background and is indicative of a robust HTS assay. Using 20 μl culture volume consistently ensured a Z′-factor ≥ 0.5 and a signal/background window > 3 while the Z′-factor for 10 μl volume was lower than 0.5 (Fig. 3A), indicating that the assay is more robust when animals are in 20 μl cultures, independent of whether signal was detected using single or orbital average read mode. In subsequent experiments with 384-well plates, animals were dispensed in 20 μl.

Fig. 3B shows a comparison of dispensing animals either manually or mechanically, using either a Multidrop Combi Reagent dispenser or a Matrix Wellmate. The S/B ratios > 2.46 indicate that GFP reporter gene activation was observed in all sinefungin treated samples. Multidrop Combi Reagent dispenser and Pherastar FS microplate reader in orbital averaging read mode was the only combination that resulted in a Z′-factor value ≥ 0.5 (0.51), and was therefore used in subsequent experiments.

**Fig. 4:**
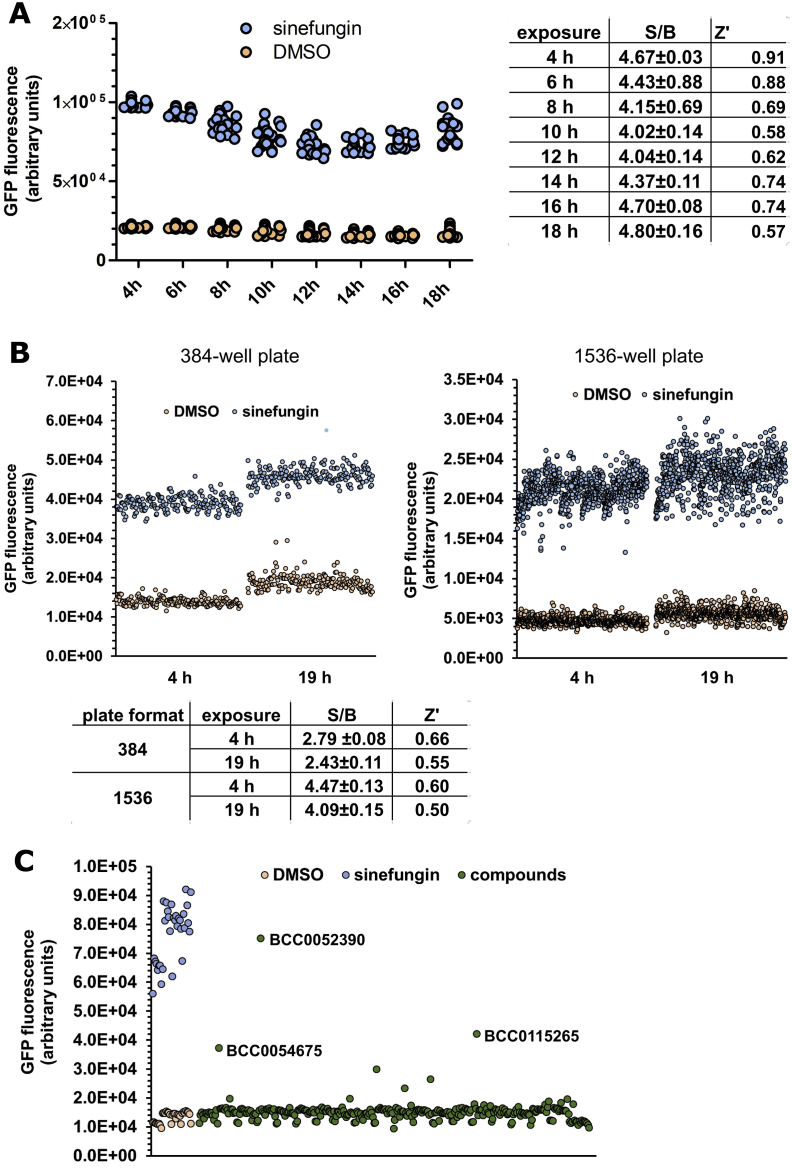
**Treatment with sinefungin produces a robust signal that can be detected for at least 19 h and scaled up for high-throughput screening.** A. Time course. PE796 animals were grown, dispensed and treated with 800 μM sinefungin/2% DMSO (24 wells) or 2%DMSO (24 wells) as described. The graph shows fluorescence measurements at the indicated times after compound addition for the treatment with sinefungin/DMSO and for DMSO only. B. Assays in 384- and 1536-well plates. Animals in 192 or 796 wells were either treated with 800 μM sinefungin/2% DMSO or with DMSO only and GFP fluorescence was measured after 4 h and 19 h of incubation. The graphs show the measurements taken after 4 h and 19 h. Signal to background (S/B) ratios with standard deviations and Z′-factors were calculated as described. C. Pilot screen of drug-like compounds. PE796 animals were dispensed into 384-well plates in the presence of OP50 bacteria. On each plate, 32 wells were treated with 2% DMSO/800 μM sinefungin, and 32 wells with 2% DMSO. Animals in the remaining wells were treated with 50 μM compound/2% DMSO, and fluorescence was analysed after 4 h exposure. The graph shows fluorescence caused by control treatments with sinefungin/DMSO and with DMSO only, and with compounds from the BioAscent Compound Cloud from a typical 384-well plate. BCC0052390, BCC0054675 and BCC0115265 were further investigated.

To establish the ideal workflow for the HTS, we then investigated the time-frame over which a robust signal can be observed. A previous analysis using fluorescence microscopy indicated that *gfp* reporter gene expression was detectable after about 4 h of sinefungin treatment. PE796 worms were exposed to sinefungin for a period of up to 18 h, during which fluorescence was measured at regular intervals (Fig. 4A). At all time points the S/B ratio was similar and between 4.02 and 4.80. The Z′-factor was around 0.9 at the early time points, and was reduced at later times, although still clearly > 0.5, indicating that the assay is robust over the 4–18 h time window.

To test the consistency of the assay we scaled up the number of wells representing positive and negative controls, with 192 wells with sinefungin-treated animals and 192 wells DMSO control-treated animals (Fig. 4B). The S/B ratios of 2.79 and 2.43 reflect the clear activation of the reporter gene in response to the addition of sinefungin at both 4 h and 19 h time points, and Z′-factors of ≥ 0.55 confirm the robustness of the assay.

We also tested whether the assay can be adapted to the 1536-well plate format. 5–10 animals in 10 μl medium were dispensed into each well, and then 768 wells were treated with sinefungin/DMSO and 768 wells with DMSO only (Fig. 4B). The S/B ratios of 4.09 and 4.47 indicated robust signal detection in wells treated with sinefungin, and Z′-factors of ≥ 0.50 indicate that the assay can also be executed using the 1536-well plate format.

These experiments also indicate that while prolonged incubations are possible, this assay is more robust with shorter incubation times. The increase in the error associated with replicates and the resulting reduction in Z′-factor over longer incubation is very likely linked to evaporation occurring in wells along edges of the plate, which was observed by eye. To reduce the effect of evaporation, assay plates were incubated in sealed containers humidified by inclusion of distilled water-soaked tissues.

We performed a pilot screen of 6775 small molecules from the BioAscent Compound Cloud of diverse lead like structures, from the NIH Clinical Collection and cherry picked from the SelleckChem libraries collection. Because of the library formulation, in combination with the detrimental effect on *C. elegans* of DMSO concentration in excess of 2% (Katiki et al., 2011), the highest compound concentration we could perform the screen at was 50 μM. We confirmed that exposing PE796 to 40 μM sinefungin does result in reporter gene activation and the inhibition of *gfp* and *rps-3* SL1 *trans*-splicing (data not shown). The screen was done in 384-well plates with PE796 animals, and OP50 bacteria were included as food source. Each plate also included PE796 animals treated with sinefungin/DMSO and DMSO only. The results from one 384-well plate, testing components of the BioAscent Compound Cloud, are shown as example in Fig. 4C. 68 compounds had a ≥ 20% effect relative to sinefungin/DMSO control treated animals, and were further investigated. Three of these, BCC0054675, BCC0052390 and BCC0115265 are shown in Fig. 4C. Of these 68, 29 were eliminated because of their known fluorescent properties. 39 compounds were further analysed by dose-response assays, including the three compounds from Fig. 4C. 23 of these compounds, including BCC0052390 and BCC0054675, did not produce a dose-dependent increase in fluorescence and were therefore not further analysed. Visual inspection showed that 11 of the 16 remaining compounds were fluorescent and were therefore not further pursued. Five compounds (Cloroxine, Risperidone, Nitazoxanide, BCC0100201 and BCC0115265) were re-tested by high resolution fluorescence microscopy. Treatment with up to 160 μM compound did not produce the typical intestinal *gfp* fluorescence produced for example by sinefungin (Fig. 2), ruling these compounds out as inhibitors of SL1 *trans*-splicing. This screen was repeated in the absence of OP50 bacteria, and similarly failed to identify any hit compounds. The screening of this limited number of compounds did not lead to the identification of any hits. However, the ease with which we were able to rule out compounds as *bona fide* inhibitors of SL1 *trans*-splicing supports the suitability of our *gfp*-reporter assay for the detection of inhibitors of SL1 *trans*-splicing.

### 3.3 Modification of *C. elegans* culture conditions

**Fig. 5:**
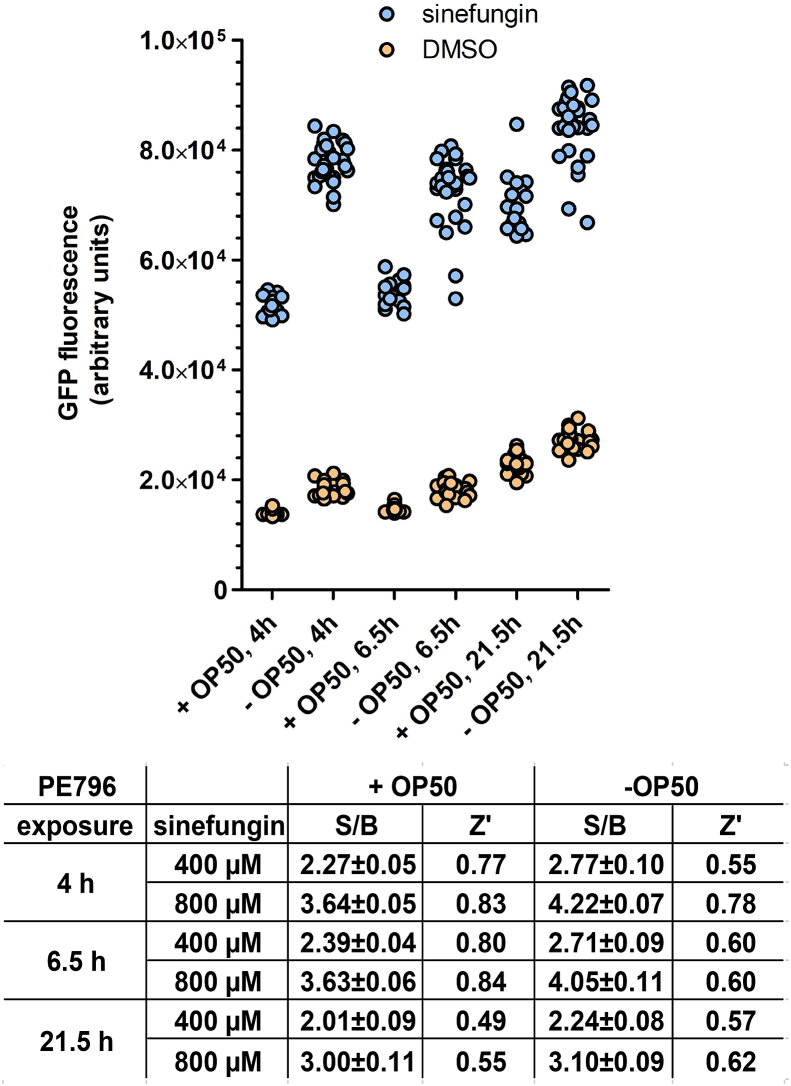
**Sinefungin-induced reporter gene activation is similar in the presence and absence of *E. coli* OP50.** PE796 animals were dispensed with or without OP50 *E. coli* and treated with compounds as described. GFP fluorescence was measured at the indicated times after sinefungin addition. The graph shows fluorescence measurements for treatments with 800 μM sinefungin/2% DMSO (16 replicates each) and with 2% DMSO only (32 replicates) at the indicated times. The table summarises signal/background ratios (S/B) with standard deviations and Z′-factors for treatments with 400 μM and 800 μM sinefungin compared to the DMSO treatment control.

The experiments described in Fig. 3, Fig. 4 were performed with incubations that contained OP50 *E. coli*. To determine whether the inclusion of the bacteria, which could interfere with the assay by for example metabolising compounds (Luo et al., 2009), is necessary, we compared the assay performance in the presence and absence of bacteria (Fig. 5). PE796 animals were treated with 400 μM and 800 μM of sinefungin and DMSO, or with DMSO only, and GFP expression was measured after 4 h, 6.5 h and 21.5 h. S/B ratios were similar for treatments with/without OP50 bacteria, and although Z′-factor values were generally lower in treatments without OP50, they were ≥ 0.50, indicating that the omission of bacteria does not affect the HTS quality of the assay.

### 3.4 Genetic modification of *C. elegans* increases robustness of the high-throughput screening assay

The cuticle of *C. elegans,* which is one of the entry points for drugs, is an extracellular matrix that lines the exterior, oral and rectal cavities, and acts as an exoskeleton. The major component is collagen, and other components are cuticulin, lipids and glycoproteins. The cuticle is relatively impermeable to chemical compounds (Cox et al., 1981; Page and Johnstone, 2007), thus treatments or mutants that increase the permeability of the cuticle could increase the entry of drugs and reduce the effective concentration required, increasing the sensitivity during the screening process.

The *bus-8* gene is required for epidermal integrity and cuticle surface formation (Partridge et al., 2008). Several mutant alleles of this gene have been isolated that affect the structure of the cuticle, including the *e2698* allele (Gravato-Nobre et al., 2005). *bus-8 (e2698)* mutants are phenotypically normal in terms of appearance and movement but have a high permeability for various small molecules (Partridge et al., 2008). Also, *bus-8 (e2698)* mutants have been previously used in the screening of drug libraries (McCormick et al., 2013).

**Fig. 6:**
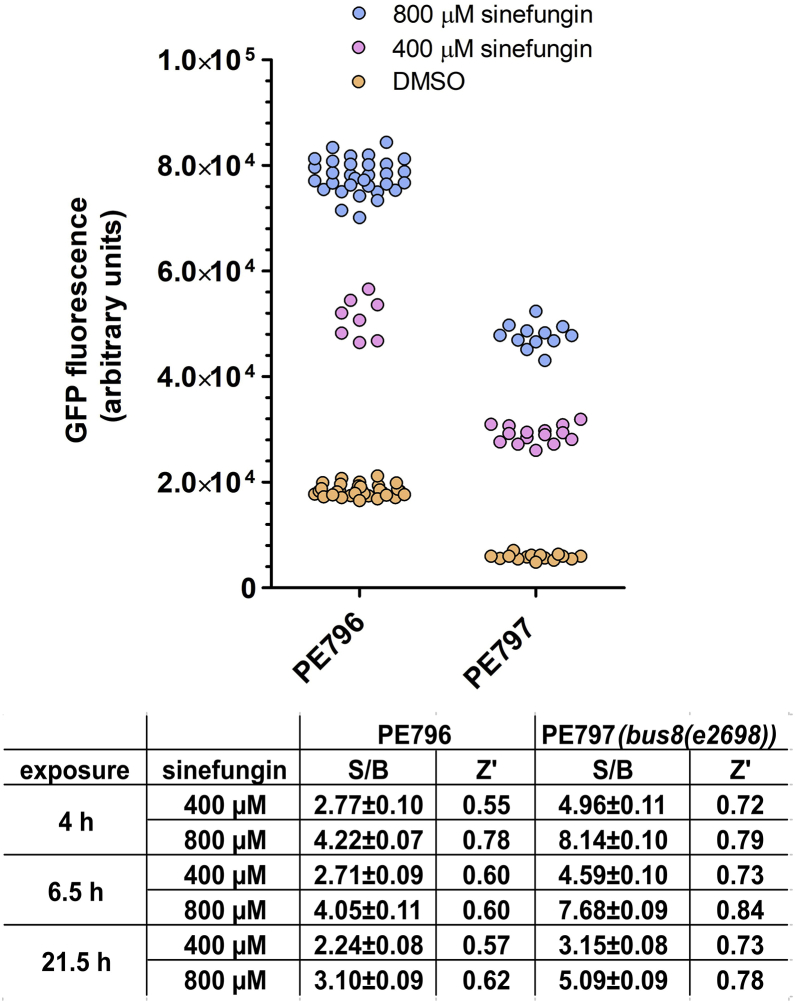
**The *bus-8* mutation affecting cuticle integrity increases high-throughput screen robustness.***bus-8*^(+)^ and *bus-8*^(−)^ animals (PE796 and PE797) animals were grown, dispensed and treated with sinefungin/DMSO or DMSO only in the absence of OP50 bacteria. GFP fluorescence was measured at the indicated times after the addition of compounds. The graph shows fluorescence measurements 4 h after addition of compounds. Normally 12 or 15 wells were measured for incubations with PE797 and 32 for incubations with PE796 except for the treatments of PE796 with 400 μM sinefungin, where 8 wells were measured. The table shows signal/background ratios (S/B) with standard deviations and Z′-factors for 4 h, 6.5 h and 21.5 h treatment with compounds. The values for PE796 animals are as in Fig. 5 without OP50 bacteria. Note that the assay is more robust with *bus-8*^(−)^ PE797 animals. The reduced absolute levels of GFP fluorescence in these animals may be linked to the different genetic background of this strain.

We produced PE797, a cuticle defective transgenic *C. elegans* strain with the *bus-8 (e2698)* allele and the spliced leader *trans*-splicing GFP reporter construct. PE797 and PE796 animals were exposed to 400 μM and 800 μM sinefungin/DMSO or to DMSO only, and GFP fluorescence was measured after 4 h, 6.5 h and 21.5 h (Fig. 6). Treatment of PE797 animals with sinefungin produced a stronger signal with statistically significantly higher S/B ratio compared to PE796 animals at all time points and sinefungin concentrations (One-way ANOVA, p < 0.01). This was also reflected in the higher Z′-factor values obtained with PE797 animals. This is compatible with the cuticle being a significant obstacle to small compounds, and identifies a strategy to enhance the sensitivity of the assay, by increasing cuticle permeability.

In conclusion, we have shown that treatment of *C. elegans* with sinefungin affects development and reduces fecundity. We have also shown that sinefungin acts as an inhibitor of SL1 *trans*-splicing in *C. elegans,* however it is not clear whether these two effects of sinefungin treatment are linked. Using sinefungin, we have developed a robust, GFP-based high-throughput screening assay protocol that is selective for the identification of compounds that inhibit SL *trans*-splicing. A pilot screen of 6775 compounds has identified a small number of candidate positives which were identified as false positives in repeat tests. This gives confidence that the number of false positives during screening of other compound libraries will be manageable. We have further identified modifications of the assay, by changing culture conditions and using *C. elegans* strains with a more permeable cuticle, that enhance the versatility and sensitivity of this screen.

Here we have used a microplate reader to detect GFP fluorescence. The assay is also suitable for analysis by high content imaging. This would allow us to eliminate autofluorescent compounds at an early stage and has the added advantage to allow direct visualisation of any effects on *C. elegans* morphology and behaviour.

## Competing interest

The authors declare that they have no competing interest. The funding agency played no role in the design or implementation of the study, analysis or interpretation of the data, or the preparation and submission of the manuscript.

## Authors′ contributions

JP, BM and BC conceived and designed the experiments. GP and AA performed the experiments. GP, MS, SM, JP, BM, BC analysed the data. SM and MS contributed reagents, analysis tools, access to screening equipment and provided training. LP contributed reagents and training. BM, GP, BC and JP wrote the manuscript. All authors read and approved the final manuscript.
